# Risk Factors for Long COVID in Older Adults

**DOI:** 10.3390/biomedicines11113002

**Published:** 2023-11-08

**Authors:** Yunguang Hu, Yifan Liu, Huiwen Zheng, Longding Liu

**Affiliations:** Key Laboratory of Systemic Innovative Research on Virus Vaccines, Institute of Medical Biology, Chinese Academy of Medical Sciences and Peking Union Medical College, Kunming 650118, China; hyg0320@163.com (Y.H.); liuyifan@imbcams.com.cn (Y.L.); zhenghuiwen12@126.com (H.Z.)

**Keywords:** long COVID, elderly, COVID-19, immunosenescence, risk factors, public health

## Abstract

As time has passed following the COVID-19 pandemic, individuals infected with SARS-CoV-2 have gradually exhibited a variety of symptoms associated with long COVID in the postacute phase of infection. Simultaneously, in many countries worldwide, the process of population aging has been accelerating. Within this context, the elderly population has not only become susceptible and high-risk during the acute phase of COVID-19 but also has considerable risks when confronting long COVID. Elderly individuals possess specific immunological backgrounds, and during the process of aging, their immune systems can enter a state known as “immunosenescence”. This further exacerbates “inflammaging” and the development of various comorbidities in elderly individuals, rendering them more susceptible to long COVID. Additionally, long COVID can inflict both physical and mental harm upon elderly people, thereby reducing their overall quality of life. Consequently, the impact of long COVID on elderly people should not be underestimated. This review seeks to summarize the infection characteristics and intrinsic factors of older adults during the COVID-19 pandemic, with a focus on the physical and mental impact of long COVID. Additionally, it aims to explore potential strategies to mitigate the risk of long COVID or other emerging infectious diseases among older adults in the future.

## 1. Introduction

As of 26 October 2023, the World Health Organization (WHO) has reported 771,549,718 confirmed cases of COVID-19 worldwide since the outbreak of SARS-CoV-2 in February 2020 [1]. COVID-19 not only causes a series of acute pathological responses in the human body [2] but has also gained attention due to its postinfection sequelae, commonly referred to as long COVID or postacute COVID-19 syndrome. Scientists have been studying long COVID since April 2020, as seen in the LitCovid topic “Long COVID” on PubMed [3,4].

Long COVID is “a condition that occurs in individuals with a history of probable or confirmed severe acute respiratory syndrome coronavirus 2 (SARS-CoV-2) infection, typically occurring three months after the onset of COVID-19 symptoms, and lasting for at least two months without any other diagnosis to explain it. Common symptoms include fatigue, shortness of breath, cognitive dysfunction, and others; moreover, it generally affects daily functioning” [5]. The National Institute for Health and Care Excellence (NICE) defines it as “signs and symptoms that develop during or after an infection consistent with COVID-19 which continue for more than 12 weeks and are not explained by an alternative diagnosis” [6]. In a meta-analysis that included 15 studies and 47,910 patients [7], Lopez-Leon et al. reported that long COVID may manifest with as many as 50 or more different clinical symptoms. Among these symptoms, the most common were fatigue (58%), headache (44%), attention disorder (27%), hair loss (25%), dyspnea (24%), cough (19%), joint pain (19%), anxiety (13%), digestive disorders (12%), pain (11%), renal failure (1%), and PTSD (1%). Long COVID symptoms affect multiple organs and systems within the human body.

According to current research [8], the prevalence of long COVID is estimated to be between 31% and 69%, indicating that over 200 million individuals worldwide may experience long COVID symptoms. While some studies have suggested that older adults may not be at a higher risk of long COVID than younger individuals [9], this may be because there are more younger COVID-19 survivors, and the epidemiological statistics for long COVID in elderly people exclude a substantial number of fatalities and may introduce bias towards older adults [10]. Additionally, many mechanisms of long COVID remain unclear, and there is a lack of targeted and effective treatment options [11]. Considering that older adults are a population that requires considerable health care resources, long COVID in this population remains a major challenge in public health, clinical medicine, and basic medical research.

This review summarizes the infection characteristics and intrinsic factors of older adults during the COVID-19 pandemic, with a focus on the physical and mental impact of long COVID. Additionally, it aimed to explore potential strategies to mitigate the risk of long COVID or other emerging infectious diseases among older adults in the future.

## 2. Characteristics of Older Adults Infected with COVID-19

### 2.1. Common Symptoms of COVID-19 in Elderly People

Currently, substantial research from around the world indicates that age is a significant risk factor for severe COVID-19 and adverse health outcomes [12]. In elderly individuals, common clinical symptoms of COVID-19 following SARS-CoV-2 infection include cough, fever, shortness of breath, dyspnea, myalgia, anxiety, depression, anosmia, and ageusia. Additionally, atypical symptoms such as neurological manifestations, elevated white blood cell counts, and elevated muscle enzyme levels are more prevalent among elderly individuals. Furthermore, as age increases, the probability of adverse clinical outcomes also escalates [13]. Undoubtedly, aging is an important risk factor for severe COVID-19 and its adverse health consequences, including hospitalization, ICU admission, and mortality [12].

### 2.2. Prolonged Viral Shedding

Since the outbreak of SARS-CoV-2, several reports have highlighted that the risks of hospitalization, ICU admission, and death increase with age [14,15,16,17,18], indicating that older adults were a high-risk group during the COVID-19 pandemic. During the acute phase of COVID-19, older adults exhibit distinct characteristics compared to younger age groups. The transmission of the virus depends on the shedding of infectious viral particles, which is influenced by both viral and host factors [19]. According to Chen et al., the median duration of SARS-CoV-2 RNA shedding is generally approximately 12 days, and as age increases, the median duration of viral shedding also increases. The median durations for patients under 16 years, 16–49 years, 50–64 years, and over 65 years old were 8.0 (6.0–15.0), 11.0 (7.0–15.0), 13.0 (10.0–17.0), and 13.0 (10.0–19.0) days, respectively. Advanced age is independently associated with prolonged shedding of SARS-CoV-2 RNA in the respiratory tract [20]. Although different studies report varying median times of viral RNA shedding, all demonstrate an association between older age and prolonged shedding of the virus [21,22,23]. Some studies have indicated that older patients (over 60 years) have a significantly higher proportion of continued positive SARS-CoV-2 detection in viral genome testing three weeks after initial infection compared to younger patients [24].

### 2.3. Intensive Tissue Damage Related to Inflammation in the Lung and Other Organs

**Figure 1:**
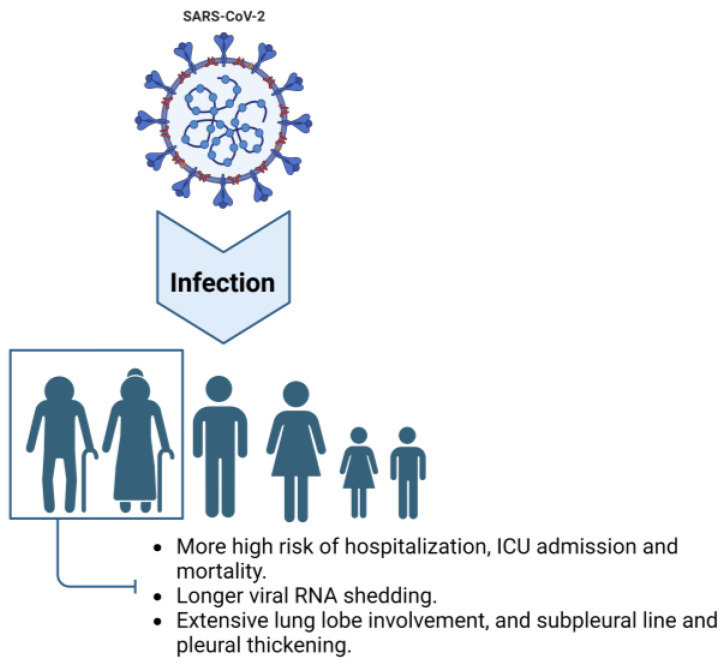
Characteristics of older adults infected with COVID-19. Compared to other age groups, elderly individuals exhibit a higher risk of hospitalization and ICU admission and a higher mortality rate when infected with SARS-CoV-2. Additionally, older adults experience longer viral shedding periods and display radiological symptoms characterized by extensive involvement of lung lobes, subpleural lines, and pleural thickening. Image created with BioRender (https://biorender.com/, accessed on 20 June 2023).

In terms of medical imaging, there are also age-related differences among COVID-19 patients. In a retrospective analysis of chest CT images from 72 COVID-19 patients, older patients had a higher proportion of extensive lung lobe involvement and were more likely to have subpleural lines and pleural thickening [25]. Another retrospective analysis of chest CT images from 50 patients revealed that interlobular septal thickening and honeycombing were more common in older individuals. Moreover, there was a statistically significant difference in the total CT score among different age groups, with the average total score being 7.3 in the older group, higher than the 3.9 in the younger group [26]. Notably, both short-term and long-term follow-up of hospitalized and recovered COVID-19 patients showed that older age was a risk factor for pulmonary fibrotic lesions [27,28]. Additionally, older adults exhibit a decreased type I interferon (IFN-I) response and increased levels of proinflammatory cytokines such as interleukin (IL)-6, IL-12, IL-1β, and tumor necrosis factor (TNF)-α after SARS-CoV-2 infection, which may exacerbate the severity of COVID-19 in this population [29] (see Figure 1).

The age-related characteristics of SARS-CoV-2 infection have also been demonstrated in experimental animal models. Studies have found that older ferrets exhibited more severe lung pathology and clinical symptoms, while older non-human primates exhibited sustained proinflammatory responses [30]. In non-human primates of advanced age, Speranza et al., Blair et al., and Zheng et al. all noted that cytokine levels emphasized sustained proinflammatory responses [31,32,33]. Older rhesus macaques have also been found to have more active viral replication in the lungs and experience more severe interstitial pneumonia caused by the virus than younger macaques [34].

## 3. Risk Factors for Developing Long COVID in Elderly Individuals

SARS-CoV-2 invades the human body and causes pathological damage in four stages: (1) invasion; (2) blockade of antiviral innate immunity; (3) interaction between viral defense mechanisms and adaptive immunity; and (4) acute or long-term complications of COVID-19 [35]. These stages highlight the importance of the immune system in SARS-CoV-2 infection, and studies have shown that the severity and mortality of the infection in older adults is closely related to the characteristics of their immune system. In young and middle-aged people, the immune system is, normally, resting but able to mount a strong but transient dynamic response promptly after detecting an “invasion”. However, during the aging process, the immune system undergoes mild, chronic activation that leads to a prolonged response time and decreased response magnitude when stimulated [36]. This state, known as immunosenescence, is characterized by chronic low-grade inflammation and a decline in the ability to respond to and defend against external threats [37,38]. As a result, older individuals often experience worsening symptoms with increasing age, atypical clinical manifestations, and a delayed fever response when encountering SARS-CoV-2 infection.

### 3.1. Immunosenescence

The immune system plays a critical role in the response to SARS-CoV-2 infection, and the characteristics of the aging immune system are closely related to the increased risk of severe illness and mortality among older individuals. Aging is associated with immunosenescence, leading to a prolonged and less effective immune response, which may contribute to the more severe outcomes observed in elderly individuals infected with SARS-CoV-2.

Immunosenescence refers to changes in immune function caused by aging [39]. It not only substantially impacts both innate and adaptive immunity but is also a risk factor for most age-related diseases [40,41,42]. In the context of innate immunity, immunosenescence is characterized by decreased phagocytic capacity of neutrophils [43], reduced microbicidal activity [44], decreased number of macrophage precursors, reduced phagocytic function [45], diminished phagocytic capacity of monocytes [46], impaired production of new natural killer (NK) cells [47,48], and decreased cytotoxicity of NK cells [49]. In terms of adaptive immunity, aging is associated with decreased production of naive B cells and a reduced ability to respond to new antigens and leads to a shorter duration of IgG production by plasma cells and compromised specific humoral immune function against pathogens and vaccines [50,51,52]. Additionally, with the atrophy and functional decline of the thymus in older individuals, there is a decline in the number of naive T cells [53], while the number of antigen-specific memory T cells and effector T cells increases with age [54]. Research has shown that many cytokines, such as IFN-γ, IL-2, and TNF-α, in CD8+ cells increase with age, and IL-4, IL-6, and IL-10 increase in the memory subset [55]. Aging also affects the quantity, subset distribution, and function of Tregs [56] and leads to dysregulation of microRNAs (miRNAs) [36], which are non-coding single-stranded RNAs (ssRNAs) that play a crucial role in immune regulation [57]. In summary, with a series of immune dysfunctions, some functions are downregulated, while others are upregulated, leading to the occurrence of widespread low-grade chronic inflammation known as inflammaging [58].

### 3.2. Chronic Inflammation (Inflammaging)

Inflammation, responding to exogenous or endogenous infections or injuries, is regulated by the immune system. When the body perceives a threat, inflammation regulated by the immune system will be activated, which subsides when the threat diminishes [59,60]. However, older individuals, due to a prolonged lifetime of exposure to various stimuli, such as chronic infections, obesity, cellular senescence, and aggregation of exogenous or endogenous macromolecules in the body [61,62], combined with immunosenescence that occurs with aging, have an elevated level of age-related proinflammatory markers even in the absence of overt clinical disease or threats. This leads to a chronic low-grade inflammatory status known as “inflammaging” [63]. Such inflammaging is a risk factor for several age-related diseases, including but not limited to hypertension [64,65,66], type 2 diabetes [64], cardiovascular diseases (CVDs) [67,68], chronic kidney disease [69], cancer [70,71], and depression [72,73].

### 3.3. Comorbidity

Comorbidity is not only a risk factor for susceptibility to SARS-CoV-2 infection [74] but also a risk factor for the severity of COVID-19 [75]. In a study of 1590 laboratory-confirmed COVID-19 patients in China, the risk ratio for patients with at least one comorbidity was 1.79 (95% CI, 1.16–2.77), and for patients with two or more comorbidities, the risk ratio was 2.59 (95% CI, 1.61–4.17) [76]. Another meta-analysis including six studies and 1527 patients indicated that 17.1% of COVID-19 patients had hypertension, 16.4% had cardiovascular diseases, and 9.7% had diabetes [77]. Comorbidities are even more common in patients with severe COVID-19, including hypertension, diabetes, COPD, cardiovascular diseases, chronic kidney disease, malignancies, and others, which can lead to severe outcomes in infected individuals [75,78,79,80,81]. Hypertension, cardiovascular disease, and diabetes were the most common comorbidities in patients who died due to COVID-19 [82]. A meta-analysis of 51 studies involving 48,317 diagnosed COVID-19 patients indicated that the prevalence of hypertension, diabetes, and cardiovascular diseases was lower among young patients than among older patients, and hypertension, diabetes, and cardiovascular diseases were significantly associated with patient mortality across all age groups [83].

**Figure 2:**
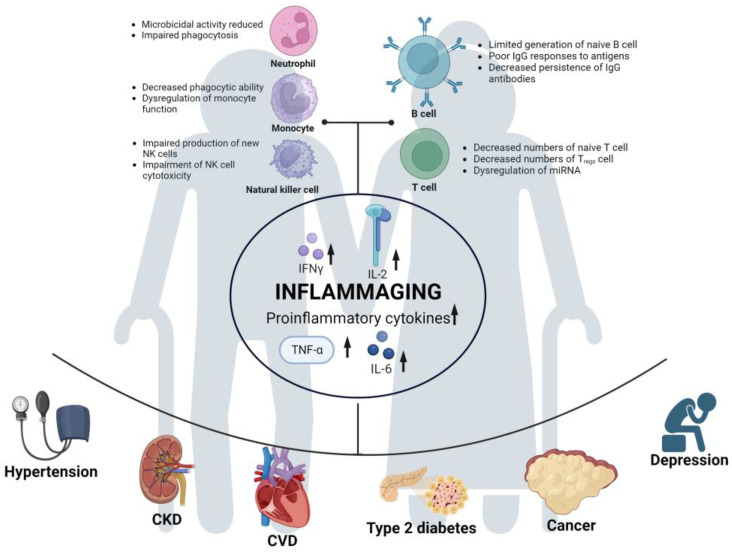
Risk factors for long COVID development in older adults. Immunosenescence in the elderly population leads to immune dysregulation, resulting in reduced or dysfunctional functions of various immune cells. Subsequently, this leads to the upregulation of various proinflammatory cytokines, giving rise to a state of “inflammaging” in the aging immune system. This chronic and long-term inflammatory state makes older adults susceptible to a wide range of comorbidities. It further increases the risk of COVID-19 and long COVID in the elderly population. Image created with BioRender (https://biorender.com/, accessed on 20 June 2023).

In summary, a cascade of events occurs in older individuals: immunosenescence leads to immune dysregulation, which results in inflammaging. This inflammaging contributes to the development of comorbidities in older individuals, increasing their vulnerability to SARS-CoV-2 infection and the severity of COVID-19 (see Figure 2). Furthermore, a higher number of SARS-CoV-2 infections can potentially increase the risk of long COVID in older individuals [84]. Additionally, the severity of COVID-19 increases the risk of long COVID. Retrospective analysis has shown that older age and severe illness are associated with a higher risk of fatigue and experiencing multiple long COVID symptoms [85]. Therefore, older adults not only face a higher risk during acute COVID-19 but also remain at a greater risk for long COVID, which can cause them physical and mental harm.

## 4. Typical Disorders in Older Long COVID Patients

### 4.1. Pulmonary Physiological Damage

“Cytokine storms” have been recognized as triggers for severe COVID-19 [86], characterized by abnormally elevated serum proinflammatory cytokine levels, including IL-6, IL-1β, IL-2, IL-8, IL-17, granulocyte colony-stimulating factor (G-CSF), TNF, etc., leading to massive infiltration of neutrophils and macrophages in the lungs and thickening of alveolar walls [87]. Prolonged lung tissue damage may lead to excessive deposition of extracellular matrix (ECM) molecules, such as collagen and fibronectin, during tissue repair, ultimately resulting in pulmonary fibrosis.

Pulmonary fibrosis is a chronic progressive lung disease that steadily leads to lung architecture disruption and respiratory failure [88]. After fibrosis occurs in the lungs, the thickening of the alveolar walls hinders gas exchange, leading to decreased and/or declining lung function, dyspnea, fatigue, and exercise intolerance [89]. An increasing number of patients with pulmonary fibrosis have been observed, particularly among patients with COVID-19-associated acute respiratory distress syndrome (ARDS) [90,91]. Since 7.2% to 31% of COVID-19 patients have ARDS [23], pulmonary fibrosis is considered a major long-term adverse health outcome of COVID-19 [89]. Factors such as male sex, older age, smoking, and certain comorbidities (diabetes, hypertension, cardiovascular diseases, etc.) have been identified as risk factors for pulmonary fibrosis [92].

### 4.2. Unstable Cytokine Balance

Some studies have shown that long COVID patients, after experiencing the inflammatory response or even cytokine storms during the acute phase of COVID-19, do not recover to a resting level of inflammation in the short term. In the context of immunosenescence, inflammaging, and comorbidities in older individuals, this chronic dysregulation may further exacerbate the immune system imbalance, leading to worsened long COVID symptoms in older adults.

In a study by Queiroz et al. [93], cytokine analysis of 225 post-COVID-19 patients revealed that 135 patients with long COVID had higher levels of IL-17 and IL-2, while 90 patients without sequelae had higher levels of IL-10, IL-6, and IL-4. Thus, the authors suggested that elevated serum levels of IL-17 and IL-2, as well as decreased levels of IL-4 and IL-10, seemed to form the cytokine profile of long COVID. However, Williams et al. [94] found significantly reduced levels of IL-17 and IL-2, together with IL-4 and IL-6, as well as the absence of IFN-γ and IL-8, in 12 patients with long COVID compared to healthy individuals. These researchers proposed that immune depletion might be a driving factor in long COVID, as the complete absence of IFN-γ and IL-8 prevented healing in the lungs and other organs after acute infection, thus reducing the ability to resist subsequent infections and leading to various long COVID symptoms. The apparent discrepancy of IL-2 and IL-17 results in the two studies may probably be explained by the different experimental conditions. However, these observations collectively reflect the chronic dysregulation of the immune system in long COVID patients. Another study among 318 recovered COVID-19 patients discovered that 67.8% of them reported long COVID symptoms, and those reporting long COVID symptoms had elevated levels of plasma IL-1β, IL-6, and TNF [95]. Similarly, a study in Brazil found that long COVID patients had elevated levels of IL-17 and IL-2, reduced levels of IL-4, and decreased parasympathetic nervous system activation, along with increased body temperature [96]. The authors suggested that the variations in serum IL-17, IL-4, and IL-2 seemed to represent characteristic cytokine features of long COVID, which could potentially serve as therapeutic and preventive targets. Another preliminary study from Ireland also indicated that, compared to healthy controls, recovered COVID-19 patients had elevated plasma levels of proinflammatory cytokines (e.g., IL-6, IL-8, IL-15, and IL-1RA) [97]. Overall, these studies on cytokines in long COVID patients indicate that the inflammatory response in their bodies has not returned to normal levels. 

### 4.3. Long Period of Fatigue

Various mechanisms related to long COVID have been proposed, among which the most crucial may be the mechanism related to fatigue, which is the most frequently reported clinical symptom of long COVID and potentially the symptom that has the greatest impact on elderly individuals. The health-related quality of life (HRQoL) is an essential indicator for evaluating the impact of the disease and disability on different dimensions of health [98]. The impact of fatigue on HRQoL of elderly individuals may be greater than the overall impact of other symptoms [99]. Fatigue can have a significantly negative effect on physical and functional activity, leading to a series of vicious cycles of physical weakness, muscle wasting, and reduced cardiopulmonary function in elderly people [100]. Fatigue is already a major health problem in elderly people [101], and the new fatigue symptoms that appear in long COVID—resembling chronic fatigue syndrome [102]—may exacerbate the condition, leading to greater muscle and cardiopulmonary function decline and making elderly people more susceptible to chronic diseases [99,103]. Chronic fatigue syndrome may also be associated with a range of symptoms, including pain, depression, sleep disorders, and emotional disorders [104,105], substantially reducing the quality of life of elderly individuals and requiring more medical resources.

### 4.4. Microthrombi and Endothelial Dysfunction

Another mechanism that may cause fatigue in long COVID patients is the formation of microthrombi and endothelial dysfunction. ACE2, the primary receptor for SARS-CoV-2, is highly expressed in the endothelium of small arteries, large arteries, and veins in the human body [106], suggesting that SARS-CoV-2 may attack not only the respiratory system but also the blood vessel endothelium in major organs [107]. Damage to the blood vessel endothelium can lead to activation and aggregation of clotting factors/thrombin, which may directly cleave the spike protein S1 of SARS-CoV-2, enhancing its infectivity during the entry phase [108] and further causing damage to the blood vessel endothelium. Endothelial damage can also cause coagulation and microthrombi formation, which may lead to systemic dysfunction and various clinical sequelae [109]. In fact, endothelial dysfunction and coagulation dysfunction have been observed as key pathological changes in acute and postacute COVID-19, especially in severe cases [110,111]. A meta-analysis of 11 studies with follow-ups of 21 to 180 days that included 18,949 patients revealed that the incidence of venous thromboembolism among discharged COVID-19 patients ranged from 0.2% to 14.8%, with a positive correlation between incidence and age [112]. Pulmonary fibrosis, decreased lung function, endothelial dysfunction, and microthrombi formation may lead to local or systemic hypoxia. In the context of persistent SARS-CoV-2 and immunosenescence in elderly individuals, it could lead to prolonged endothelial damage, widespread endothelial inflammation, and extensive thrombosis [113], further damaging various organs and tissues and forming a vicious cycle that could severely compromise the physiological health of long COVID patients.

### 4.5. Disruption of the Human Microbiome or Virome

Elderly individuals with long COVID may have a disrupted human microbiome or virome. Humans harbor a large number of bacteria, viruses, and fungi in many parts of the body, such as the gut, respiratory tract, oral cavity, and urinary tract [114,115]. Under healthy conditions, the body maintains a balanced state with these microorganisms and viruses under the regulation of the immune system, forming the stable ecosystem of the human microbiome and virome [116]. However, infection by new pathogens can lead to immune system dysregulation or even immune system dysfunction, disrupting the stability of the microbiome and virome and leading to subsequent diseases. For example, H1N1 can weaken the host’s immune response through various mechanisms [117], leading to changes in the composition of the respiratory microbiome in H1N1-infected mice [118]. SARS-CoV-2 may disrupt the stability of the human microbiome and virome in a similar manner. Studies have found enrichment of pathogenic bacteria in the bronchoalveolar lavage fluid and gut microbiome of COVID-19 patients [119,120], indicating a disruption of the microbiome and virome in these patients. This non-steady state condition may persist during the recovery period of COVID-19 [121], potentially leading to metabolic syndrome, diabetes, cardiovascular disease, inflammatory bowel disease, chronic fatigue syndrome, Parkinson’s disease, etc. [122,123,124]. Disruption of the gut microbiome is believed to increase the permeability of the intestinal mucosal barrier, allowing bacteria and their products—including pathogen-associated molecular patterns (PAMPs), microorganism-associated molecular patterns (MAMPs)—and molecules from damaged cells as damage-associated molecular patterns (DAMPs) to enter the circulatory system, leading to inflammaging [62,125]. Such a situation might further exacerbate the immunosenescence and inflammaging states in elderly people due to long COVID, leading to further damage to the immune system and various organs or potentially making elderly people more susceptible to breakthrough SARS-CoV-2 infections, but further research is needed to confirm this.

### 4.6. Mental Damage

Anxiety appears to be one of the most common mental symptoms in both the acute and long phases of COVID-19. A study on psychological distress in recovered COVID-19 patients found that they experienced symptoms of anxiety, depression, and stress (stress responses). Researchers have also observed that age and retirement might mutually influence psychological responses [126]. Furthermore, clinical manifestations of sleep disorders, anxiety, and depression persist even six months after recovery from acute infection [127]. Another study examining the long-term trajectory of psychological symptoms in SARS-CoV-2-infected patients noted that long COVID patients initially experienced a considerable increase in depressive symptoms, and over a 22-month follow-up, the level of depression continued to rise. The anxiety symptoms of patients with long COVID were also higher than those of patients without long COVID (short COVID), and over time, the gap between the two groups widened [128].

**Figure 3:**
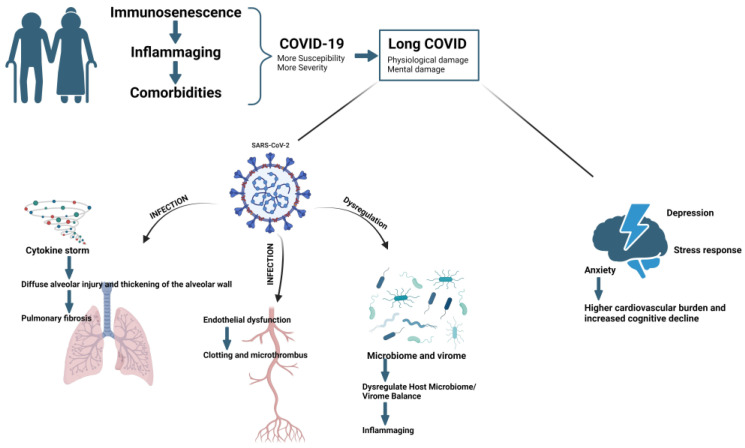
Typical disorders in older long COVID patients. In the context of aging, immunosenescence, and the presence of multiple comorbidities, the elderly population, after the acute phase of COVID-19, is also a high-risk group for long COVID. Long COVID manifests with a variety of physiological and mental symptoms in elderly individuals, considerably impacting their quality of life. Image created with BioRender (https://biorender.com/, accessed on 20 June 2023).

Multiple studies have shown that various factors related to aging, such as reduced self-confidence, decreased physical activity and mobility, loss of friends, reduced economic and physical independence, loneliness due to retirement, and chronic diseases, can make older individuals more susceptible to anxiety [129,130,131]. Anxiety in elderly people has been associated with an increased cardiovascular burden, decreased cognitive function, and a direct correlation with mortality [132], making anxiety a key factor contributing to high medical costs for individuals and society [133]. Older adults are likely to suffer both physical and mental stress from long COVID in the pandemic, which is a burden on both individuals and public health (see Figure 3).

## 5. Conclusions

Long COVID, like COVID-19, has brought both major challenges and opportunities to humanity. Never before has an acute postinfection syndrome affected such a large sample size of individuals across all age groups within such a condensed timeframe. In-depth research on long COVID in elderly individuals can not only deepen our understanding of SARS-CoV-2 and other coronaviruses’ interactions with the immune system, escape mechanisms, and breakthrough infection mechanisms but also provide insights into aging in older adults, changes in the immune system during aging, and the pathogenesis of age-related diseases.

Currently, there are various challenges in studying and assessing long COVID, including issues related to false-positive PCR tests and inaccurate antigen/antibody tests leading to biases in COVID-19 case reports, overlooking symptoms outside the respiratory system and mild cases of long COVID, and insufficient understanding of long-term symptoms following viral infections [134]. Therefore, it is essential to strengthen awareness of the hazards of long COVID, provide training and education for health care workers, educate patients about self-assessment of their health conditions and long COVID experiences, and increase investment in policies and funding for long COVID research and management [134].

To avoid being harmed by long COVID, the best approach is to prevent SARS-CoV-2 infection. Vaccination is the most effective means of preventing SARS-CoV-2 infection, and it has also been shown to reduce hospitalizations, ICU admissions, and deaths among patients of all age groups with COVID-19 [135,136,137,138,139,140]. While some studies suggest that vaccination does not significantly affect the relief of long COVID symptoms [141], a meta-analysis involving six studies and 536,291 unvaccinated patients and 84,603 vaccinated patients (before SARS-CoV-2 infection) indicated that vaccination before infection can effectively reduce the risk of long COVID [142]. Perhaps this is because vaccination can effectively reduce the risk of severe COVID-19. Therefore, vaccination before infection remains an essential measure in preventing long COVID. Given the special immune background of elderly individuals, the protective effects of vaccination may be suppressed. Inflammaging has been shown to negatively impact the response to vaccines [143,144,145]. Therefore, the development of vaccines specifically tailored for elderly people—targeting not only SARS-CoV-2 but also other pathogens, such as influenza viruses, VZV, and pneumococci, which elderly people are more susceptible to—should be prioritized. This research direction includes but is not limited to establishing relevant animal models, developing novel adjuvants, increasing antigen dosages, exploring new immunization pathways, and creating personalized vaccines [146,147,148].

Aging is a highly complex process with substantial heterogeneity, as is the immune system of older adults [149]. Each person’s lifespan and exposure to various pathogens are unique. Thus, studying the various injuries and symptoms resulting from viral acute infections in elderly people requires extensive individualized analysis, emphasizing the importance of personalized research and health care. In the establishment of future public health systems, a strong focus on the development of individual electronic medical records and public awareness—especially in middle- and low-income countries and households—can enable older individuals to conduct simple health monitoring and management on their own. This approach not only benefits the health of elderly individuals but also provides critical support for overall public health services and subsequent clinical or basic research.

## Data Availability

Not applicable.
